# Longitudinal Peripartum Assessment of Retinal Nerve Fiber Layer Thickness and Optic Nerve Sheath Diameter in Women with Preeclampsia and Normotensive Pregnant Controls

**DOI:** 10.3390/jcm15176618

**Published:** 2026-08-27

**Authors:** Raffaella L. Fantin, Franziska Huter, Alexander Franchi, Daniel Egger, Reinhard Angermann, Lisa Schlosser, Samira Abdel Azim, Katharina Knoll

**Affiliations:** 1 Department of Anaesthesiology and Critical Care Medicine, Medical University of Innsbruck, Anichstrasse 35, 6020 Innsbruck, Austria; 2 Department of Obstetrics and Gynecology, Medical University of Innsbruck, Anichstrasse 35, 6020 Innsbruck, Austria; 3 Department of Ophthalmology and Optometry, Medical University of Innsbruck, Anichstrasse 35, 6020 Innsbruck, Austria; 4 Department of Ophthalmology, Landesklinikum Mistelbach-Gänserndorf, Liechtensteinstraße 67, 2130 Mistelbach, Austria; 5 Data Lab Hell GmbH, Bahnhofstraße 15, 6170 Zirl, Austria

**Keywords:** preeclampsia, pregnancy, optical coherence tomography, optic nerve sheath diameter, retinal nerve fiber layer

## Abstract

**Background/Objectives**: Preeclampsia is associated with endothelial dysfunction and neurological morbidity. Non-invasive ocular imaging may offer insight into subclinical ocular changes associated with neurological involvement. This prospective single-center pilot study evaluated retinal nerve fiber layer (RNFL) thickness, optic nerve sheath diameter (ONSD), and angiogenic biomarkers in women with and without preeclampsia across the peripartum period. **Methods**: Pregnant women with preeclampsia and normotensive controls were enrolled at a tertiary center. RNFL thickness was assessed by optical coherence tomography and ONSD by transbulbar ultrasound at three predefined visits: antepartum, early postpartum, and late postpartum. The soluble fms-like tyrosine kinase-1/placental growth factor (sFlt-1/PlGF) ratio was determined at the antepartum visit. Group comparisons, longitudinal changes, and exploratory correlations were analyzed, with paired-eye dependence addressed using generalized estimating equations (GEE). **Results**: Thirty-eight women were included; after reclassification of three controls who developed preeclampsia, 15 women were analyzed in the preeclampsia group and 23 in the control group. RNFL thickness was numerically higher in women with preeclampsia across visits; GEE analyses accounting for paired-eye measurements showed exploratory between-group differences at the early postpartum visit. ONSD did not differ significantly between groups, and no participant had a bilateral mean ONSD > 5.8 mm. No significant between-group differences in longitudinal change from early to late postpartum were observed. Exploratory analyses did not demonstrate a consistent association between the sFlt-1/PlGF ratio and the imaging parameters. **Conclusions**: RNFL thickness was numerically higher in women with preeclampsia, whereas ONSD showed no clear separation between groups. The findings remain exploratory and do not establish a diagnostic or prognostic role for either imaging parameter. Larger prospective studies are needed to determine the clinical relevance of non-invasive ocular imaging in preeclampsia.

## 1. Introduction

Preeclampsia affects approximately 4% of pregnancies worldwide and remains a major cause of maternal and perinatal morbidity and mortality [1]. Neurological complications, including cerebral edema, seizures, and stroke, contribute substantially to adverse maternal outcomes [2]. Diagnostic options to detect early or subclinical signs of elevated intracranial pressure (ICP) during pregnancy are limited, due to contraindications, invasiveness, or restricted availability of standard neuroimaging techniques.

Point-of-care ultrasound (POCUS) measurement of the optic nerve sheath diameter (ONSD) has emerged as a non-invasive tool for ICP assessment in patients with traumatic brain injury, and spontaneous intracranial hemorrhage [3,4,5]. In obstetric anesthesia, ocular sonography has been used to evaluate suspected elevated ICP in preeclamptic patients and to monitor ICP changes following epidural blood patch administration for post-dural puncture headache [6,7,8]. However, its clinical utility remains limited by inter-operator variability and the absence of validated reference ranges in pregnant populations [9,10,11].

**Figure 1:**
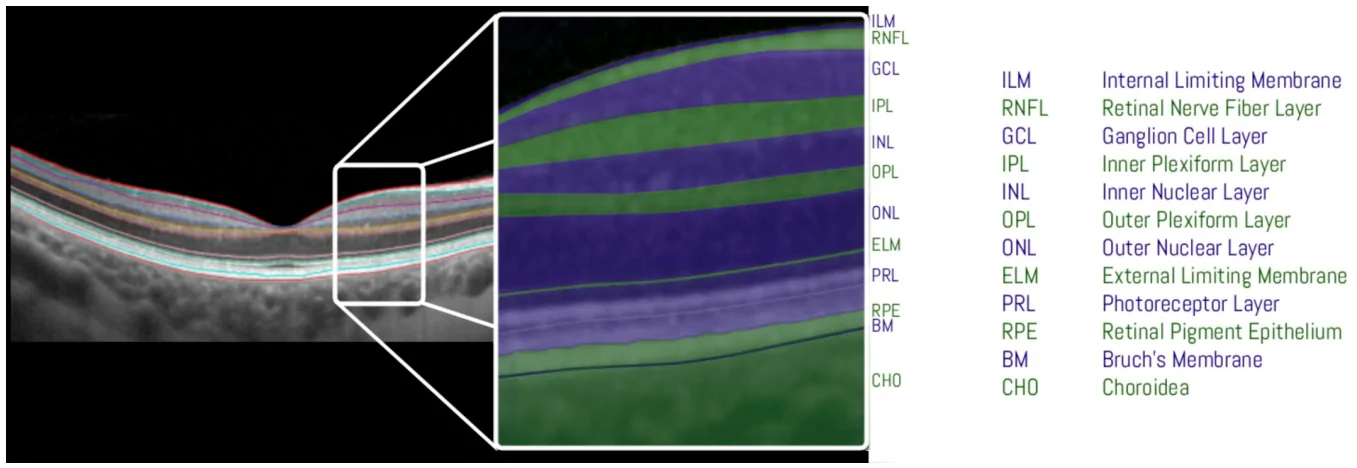
Retinal layer anatomy on spectral-domain optical coherence tomography. Original image created by the authors.

Optical coherence tomography (OCT) is a high-resolution ophthalmologic imaging technique that enables precise measurement of the retinal nerve fiber layer (RNFL), with micrometer-scale axial resolution (Figure 1) [12]. RNFL thickening may accompany papilledema, which results from optic disk swelling secondary to increased ICP and axoplasmic flow stasis in unmyelinated retinal ganglion cell axons. In preeclampsia, systemic endothelial dysfunction, altered vascular permeability, and antiangiogenic imbalance may additionally affect the retinal and optic nerve head microvasculature [13,14]. RNFL changes may therefore provide a non-invasive marker of optic nerve head changes associated with elevated ICP [15].

The soluble fms-like tyrosine kinase-1/placental growth factor (sFlt-1/PlGF) ratio reflects the angiogenic imbalance associated with preeclampsia and may provide complementary information on the relationship between systemic endothelial dysfunction and ocular structural changes [16].

Despite increasing interest in ocular biomarkers of neurological involvement in preeclampsia, no prospective longitudinal study has simultaneously evaluated OCT-derived RNFL thickness, ONSD and angiogenic biomarkers across the peripartum period.

The aim of this study was to evaluate RNFL thickness in pregnant women with and without preeclampsia using OCT at predefined time points before and after delivery. The primary objective was to test the hypothesis that RNFL thickness is increased in women with preeclampsia compared with normotensive pregnant controls.

Secondary objectives included the longitudinal assessment of RNFL thickness and ONSD and their potential associations with the sFlt-1/PlGF ratio. We hypothesized that women with preeclampsia would show greater RNFL thickness and larger ONSD values at the antepartum assessment, followed by a postpartum decline. Associations between the imaging parameters and the sFlt-1/PlGF ratio were explored without a prespecified directional hypothesis.

## 2. Materials and Methods

Pregnant participants were prospectively enrolled at University Hospital Innsbruck between April 2021 and April 2023 after written informed consent and approval by the local Ethics Committee (1436/2020). The study complied with the Declaration of Helsinki and was reported in accordance with STROBE.

### 2.1. Participants

Eligible participants were adult pregnant women with preeclampsia or normotensive pregnancies of comparable gestational age. Women younger than 18 years, those with known optic nerve or macular pathology, and those with high myopia (>6 diopters) were excluded. Preeclampsia was diagnosed according to the American College of Obstetricians and Gynecologists (ACOG) criteria and categorized as early-onset (<34 weeks) or late-onset (≥34 weeks) [17]. Women were eligible irrespective of neurological or visual symptoms.

Women with preeclampsia received guideline-based clinical management according to institutional standards. Maternal blood pressure was closely monitored, and antihypertensive treatment was initiated or adjusted as clinically indicated. Maternal and fetal surveillance was continued until delivery. Delivery was indicated in cases of maternal deterioration, including treatment-resistant severe hypertension or hemolysis, elevated liver enzymes, and low platelet count (HELLP) syndrome, or for fetal indications, including fetal growth restriction meeting criteria for delivery or evidence of fetal compromise. When delivery before 34 + 0 weeks of gestation was anticipated, antenatal corticosteroids for fetal maturation were administered whenever clinically feasible, consisting of two intramuscular doses of 12 mg betamethasone given 24 h apart. In cases of imminent delivery before 32 + 0 weeks, intravenous magnesium sulfate was administered for fetal neuroprotection according to institutional clinical practice.

Participants underwent standardized ophthalmologic and clinical evaluations at three predefined study visits: antepartum (visit 1), early postpartum (visit 2), and late postpartum (visit 3). At each visit, blood pressure measurement, urine dipstick testing, and routine laboratory values were obtained. The sFlt-1/PlGF ratio was determined at visit 1.

Demographic, clinical, obstetric, and neonatal data, including age, prior medical history, mode of delivery, and neonatal outcomes, were collected from electronic health records (KIS PowerChart version 2018.01 (Oracle Cerner, Kansas City, MO, USA)).

### 2.2. Ophthalmologic Assessment

The ophthalmologic assessment included best-corrected visual acuity testing, non-contact tonometry, and fundoscopy. Intraocular pressure (IOP) was assessed as part of the routine ophthalmologic examination; however, individual IOP values were not prospectively recorded in the study dataset. Peripapillary RNFL thickness was obtained with spectral-domain OCT (Spectralis HRA + OCT; Heidelberg Engineering GmbH, Heidelberg, Germany) at predefined scan diameters of 3.5 and 4.1 mm centered on the optic disk [18]. All scans were acquired, analyzed and quality-controlled using Heyex 2.4.1 software (Heidelberg Engineering GmbH, Heidelberg, Germany) by trained ophthalmologists following a standardized protocol. Only scans with a signal strength above the manufacturer-recommended threshold were included in the analysis. Automated RNFL segmentation was reviewed for each scan. Scans with major segmentation errors or motion artifacts were excluded or manually corrected when feasible.

Transbulbar ONSD was acquired with a 10 MHz linear probe (Eye Cubed, Ellex Medical Pty Ltd., Adelaide, Australia), with measurements taken 3 mm behind the globe in transverse and longitudinal views. A bilateral mean ONSD > 5.8 mm was regarded as suggestive of ICP > 20 mmHg [7,19]. All ophthalmologic assessments were performed by trained investigators using calibrated equipment and predefined protocols. The ophthalmic assessors were blinded to clinical group allocation and laboratory results, including the sFlt-1/PlGF ratio.

### 2.3. Statistical Analysis

Continuous and ordinal variables, including cross-sectional measurements at each visit, were compared between the preeclampsia and control groups using Student’s *t*-test for normally distributed variables or the Mann–Whitney U test otherwise, as appropriate. The same approach was used for comparisons between early- and late-onset preeclampsia. Categorical variables were compared using Pearson’s chi-square test or Fisher’s exact test, as appropriate. Distributional assumptions were assessed using the Shapiro–Wilk test. As this study was designed as an exploratory pilot investigation, no formal a priori sample-size calculation was performed.

To account for the non-independence of paired eye measurements from the same participant, RNFL thickness and ONSD were additionally analyzed using Generalized Estimating Equations (GEE) with a Gaussian family, an exchangeable working correlation structure, participant identity as the clustering unit, eye (right/left) as a covariate, and robust sandwich standard errors. As a sensitivity analysis, group comparisons were repeated using one randomly selected eye per participant across 500 independent random selections to assess the stability of findings independent of eye selection. The individual within-participant change in RNFL thickness and ONSD from the early postpartum (visit 2) to the late postpartum (visit 3) assessment was calculated and compared between the preeclampsia and control groups using Student’s *t*-test or the Mann–Whitney U test, as appropriate (Supplementary Table S2). To account for the paired-eye structure in this longitudinal comparison, the analysis was additionally repeated using GEE models with the same clustering and correlation structure as for the cross-sectional comparisons, together with a one-eye-per-participant sensitivity analysis (500 draws) (Supplementary Table S1). Visit 1 represented a distinct antepartum physiological state and was therefore not included in this postpartum comparison. In an additional exploratory analysis, associations between baseline body mass index (BMI) and individual RNFL change from visit 1 to visit 3 were assessed using GEE models with the same clustering and correlation structure described above and Benjamini–Hochberg adjustment across the two RNFL measurement diameters. A one-eye-per-participant sensitivity analysis using Spearman’s rank correlation was repeated across 500 random eye selections.

Spearman’s rank correlation was used to assess associations between RNFL thickness, ONSD, and angiogenic markers (sFlt-1/PlGF ratio). Given the exploratory, hypothesis-generating nature of the subgroup, correlation, and BMI analyses, and the large number of comparisons performed within each, Benjamini–Hochberg false discovery rate (FDR)-adjusted q-values are reported alongside nominal (unadjusted) *p*-values for these analyses. Findings are interpreted with reference to the adjusted q-values, while nominal *p*-values are provided for descriptive completeness and comparability with prior literature. A two-sided nominal *p*-value < 0.05 was considered statistically significant for analyses not subject to FDR correction. For the subgroup, correlation, and BMI analyses, statistical significance was defined as q < 0.05 after FDR correction. Statistical analyses were performed using SPSS version 28.0 (IBM Corp., Armonk, NY, USA), GraphPad Prism version 10 (GraphPad Software, Boston, MA, USA), and R version 4.5.3.

## 3. Results

**Figure 2:**
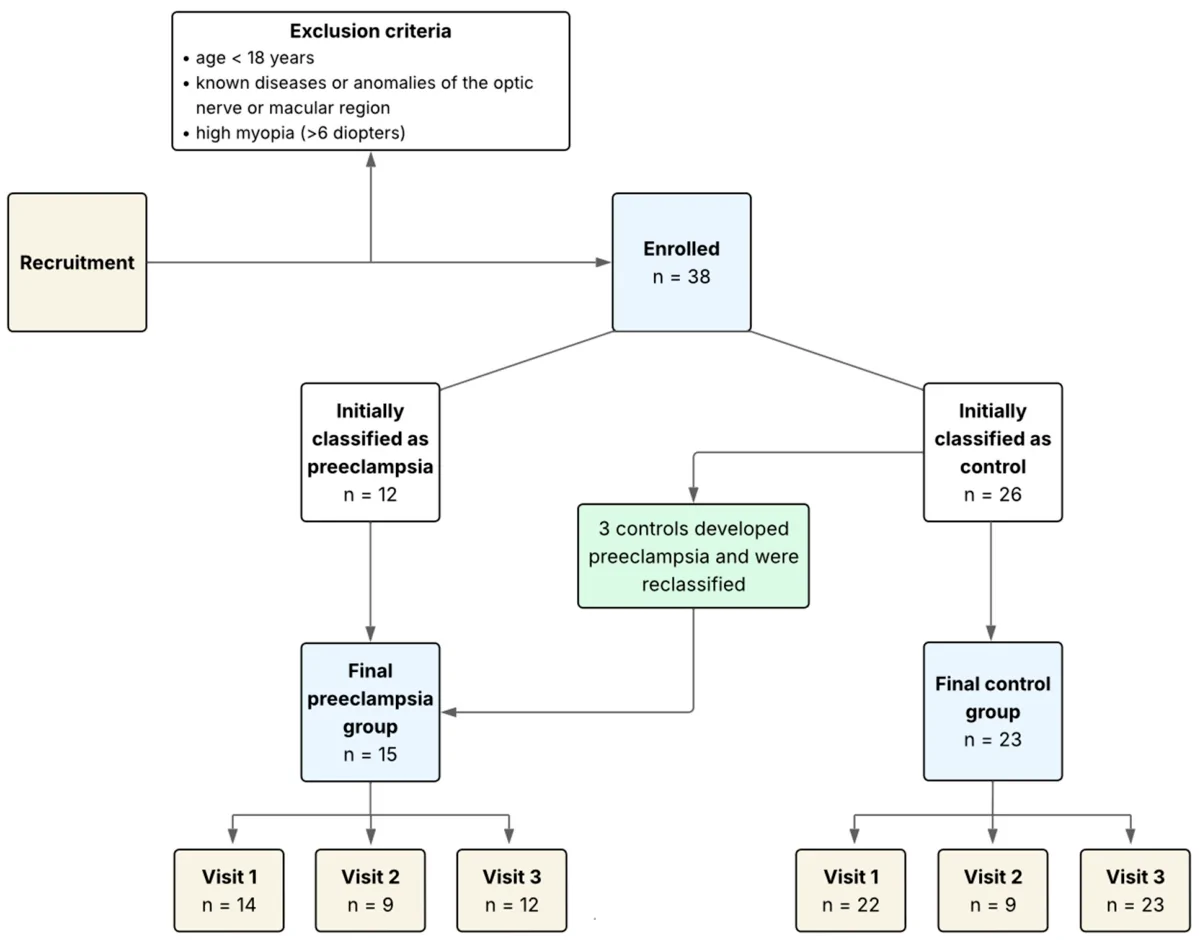
Participant enrollment, reclassification according to American College of Obstetricians and Gynecologists (ACOG) preeclampsia criteria, and availability at each study visit. Participant numbers shown reflect retinal nerve fiber layer (RNFL) data availability; optic nerve sheath diameter (ONSD) availability differed slightly and is reported in the Table 3 footnote.

A total of 38 women (76 eyes) were enrolled, of whom 12 women (24 eyes) were initially diagnosed with preeclampsia (P group) and 26 women (52 eyes) served as normotensive controls (C group). During the study, three participants in the control group developed preeclampsia (Figure 2). The patients were reclassified and analyzed accordingly. Maternal baseline and demographic characteristics are shown in Table 1 and clinical, obstetric, and neonatal characteristics in Table 2. Mean age was 30.1 (SD 5.6) years in the preeclampsia group and 32.3 (SD 4.4) years in controls. Six preeclampsia cases were early-onset and nine late-onset. At visit 1, five women with preeclampsia reported headache and one reported visual disturbances. Fundoscopy showed no retinopathy, retinal hemorrhage, papilledema, or retinal/macular edema.

Data are presented as mean (standard deviation), median [interquartile range], or *n* (%) as appropriate. *p*-values were calculated using Student’s *t*-test or the Mann–Whitney U test, as appropriate, for continuous or ordinal variables and the chi-square test or Fisher’s exact test, as appropriate, for categorical variables. ASA = American Society of Anesthesiologists; GDM = gestational diabetes mellitus; LDA = low-dose aspirin (150 mg); y = years.

Data are presented as mean (standard deviation), median [interquartile range], or *n* (%) as appropriate. *p*-values were calculated using Student’s *t*-test or the Mann–Whitney U test, as appropriate, for continuous or ordinal variables and the chi-square test or Fisher’s exact test, as appropriate, for categorical variables. CD = cesarean delivery.

### 3.1. OCT and ONSD Measurements

Table 3 summarizes the RNFL thickness and ONSD measurements across the three study visits. ONSD did not differ significantly between groups, and no participant had a bilateral mean ONSD > 5.8 mm.

**Table 3:** RNFL thickness and ONSD measurements across the three study visits.

Measurement	Timepoint	Total	Preeclampsia	Control	*p*-Value
RNFL thickness (µm)					
3.5 mm left	Visit 1	103.5 (97–109.8)	107 (100–108.5)	100 (97–109.5)	0.37
	Visit 2	103.5 (97–109.8)	107 (100–118)	97.5 (94.3–106.8)	0.09
	Visit 3	100 (96–107)	104 (101–110)	99 (95–106.8)	0.05
4.1 mm left	Visit 1	86 (82.5–93.5)	89 (85–94.5)	85 (82–92.3)	0.24
	Visit 2	87 (83.5–94)	89 (87–100)	84 (80.25–90)	0.05
	Visit 3	85 (83.3–93)	87.5 (85–94.5)	85 (83–91.8)	0.14
3.5 mm right	Visit 1	103.5 (99–108.5)	108 (99.9–113.5)	100 (98–106)	0.08
	Visit 2	107 (98.25–114.5)	110 (103–116)	99 (98–108)	0.11
	Visit 3	101 (97–108.5)	104 (98.5–112.3)	99 (96.5–105)	0.12
4.1 mm right	Visit 1	86 (82.8–93.3)	92 (84–95)	85 (82–92)	0.16
	Visit 2	88 (83–96)	95 (87–99)	84.5 (81–92.5)	0.06
	Visit 3	85 (82–93)	88.5 (82.8–94.5)	84 (82–91)	0.19
ONSD (mm)					
Left L	Visit 1	4.54 (4.04–5.1)	4.79 (4.3–5.38)	4.5 (3.91–4.92)	0.4
	Visit 2	4.72 (4.02–5.23)	4.56 (4.08–5.32)	4.72 (4.08–5.13)	0.93
	Visit 3	4.27 (4.09–4.64)	4.31 (4.18–5.1)	4.22 (4.02–4.52)	0.23
Left T	Visit 1	4.62 (4.25–5.32)	5.18 (4.57–5.77)	4.51 (4.08–4.77)	0.08
	Visit 2	4.66 (4.37–5.13)	4.66 (4.46–5.26)	4.67 (4.24–4.89)	0.8
	Visit 3	4.55 (4.27–5.03)	4.35 (4.27–4.72)	4.57 (4.26–5.32)	0.56
Right L	Visit 1	4.56 (3.9–4.96)	4.8 (4.42–5.24)	4.48 (3.83–4.86)	0.09
	Visit 2	4.38 (3.9–5.06)	4.26 (3.95–5.03)	4.38 (3.84–4.98)	1
	Visit 3	4.36 (3.98–4.84)	4.47 (3.9–4.79)	4.33 (4.05–4.85)	0.82
Right T	Visit 1	4.76 (4.21–5.11)	5.04 (4.78–5.3)	4.62 (3.94–5.07)	0.15
	Visit 2	4.39 (4.16–5.54)	4.49 (4.22–5.5)	4.38 (4.17–5.53)	0.82
	Visit 3	4.54 (4.21–5.06)	4.75 (4.37–5.09)	4.46 (4.19–4.98)	0.59

At visit 1, median gestational age was 35 + 3 weeks (32 + 3–37 + 0). The RNFL thickness at 3.5 mm was higher in the P group in both eyes. A similar pattern was observed at 4.1 mm. ONSD values were also higher in the P group. None of these differences reached statistical significance.

At visit 2 (median 2 days postpartum, IQR 1–3; RNFL: preeclampsia *n* = 9, control *n* = 9–10; see Table 3 footnote for modality-specific availability), RNFL thickness remained higher in the P group in the unadjusted per-eye analysis, although the differences did not reach statistical significance. ONSD differences between groups were also non-significant. When accounting for the paired-eye structure using GEE models with participant-level clustering, between-group differences in RNFL thickness were observed at visit 2 (3.5 mm: *p* = 0.030; 4.1 mm: *p* = 0.028), whereas differences at visits 1 and 3 did not reach statistical significance. ONSD group differences remained non-significant at all visits. As a sensitivity analysis, these comparisons were repeated using a single randomly selected eye per participant across 500 independent draws; the proportion of draws reaching nominal significance was low at Visit 1 (4.4% at 3.5 mm; 3.0% at 4.1 mm) and Visit 3 (17.8% at 3.5 mm; 0.0% at 4.1 mm), and highest at visit 2 (18.6% at 3.5 mm; 46.8% at 4.1 mm), broadly paralleling the relative strength of the GEE estimates at each visit; ONSD showed no significant difference in any draw at any visit (Supplementary Table S1).

At visit 3 (median 44 days postpartum; IQR 42–45 days), no significant between-group differences in RNFL thickness or ONSD were observed.

Data are presented as median (Q1–Q3). Group comparisons in this table are based on unadjusted per-eye analyses. Results from Generalized Estimating Equations (GEE) models accounting for the paired-eye structure with participant-level clustering are presented in the Results section and Supplementary Table S1. Number of participants with available RNFL measurements per visit: visit 1, preeclampsia *n* = 14, control *n* = 20–22 (range across eyes and scan diameters); visit 2, preeclampsia *n* = 9, control *n* = 9–10; visit 3, preeclampsia *n* = 12, control *n* = 22–23. Corresponding numbers for ONSD were: visit 1, preeclampsia *n* = 13, control *n* = 19–21; visit 2, preeclampsia *n* = 8, control *n* = 10; visit 3, preeclampsia *n* = 9, control *n* = 20–21. RNFL = retinal nerve fiber layer; ONSD = optic nerve sheath diameter; L = longitudinal; T = transverse.

### 3.2. Longitudinal Changes from Visit 2 to Visit 3

Changes in RNFL thickness and ONSD between visits 2 and 3 are summarized in Supplementary Table S2. No statistically significant differences in the degree of change were observed between the P group and C group. This was confirmed by GEE models accounting for the paired-eye structure (3.5 mm: *p* = 0.434; 4.1 mm: *p* = 0.194; ONSD longitudinal: *p* = 0.751; ONSD transverse: *p* = 0.987), and by a one-eye-per-participant sensitivity analysis, in which the proportion of 500 draws reaching nominal significance was low across all measures (RNFL 3.5 mm: 1.0%; RNFL 4.1 mm: 1.4%; ONSD: 0.0% at both orientations) (Supplementary Table S1). Exploratory analyses of baseline BMI and individual RNFL change from visit 1 to visit 3 showed no association at 3.5 mm (*p* = 0.51). At 4.1 mm, a nominal association was observed (*p* = 0.029), which did not remain significant after FDR adjustment (q = 0.058) and was not supported by a one-eye-per-participant sensitivity analysis (Spearman’s rank correlation, 500 draws), which showed no robust association at either depth (3.5 mm: median ρ = 0.155, 4.2% of draws *p* < 0.05; 4.1 mm: median ρ = 0.190, 7.0% of draws *p* < 0.05).

### 3.3. Subgroup Analysis: Early- vs. Late-Onset Preeclampsia

RNFL thickness was numerically higher in the early-onset group at all measurement sites (Supplementary Table S3), with one nominally significant difference observed at 3.5 mm in the right eye (*p* = 0.045; FDR-adjusted q = 0.24). This difference did not remain significant after adjustment for multiple comparisons. ONSD values did not differ significantly between subgroups. The sFlt-1/PlGF ratio was also numerically higher in women with early-onset than in those with late-onset preeclampsia (median 350.8 vs. 95.6), although the difference was not statistically significant (*p* = 0.06; FDR-adjusted q = 0.24).

### 3.4. Association Between Angiogenic Biomarkers and Imaging Parameters

A post hoc exploratory analysis was conducted to assess the association between the sFlt-1/PlGF ratio and RNFL thickness and ONSD measurements at visit 1 (Supplementary Table S4). In the preeclampsia group, a nominally significant positive correlation was observed between the sFlt-1/PlGF ratio and RNFL thickness at 4.1 mm in the right eye (ρ = 0.621, *p* = 0.024; FDR-adjusted q = 0.384). The association did not remain significant after FDR adjustment, and no significant associations were identified for ONSD or in controls.

## 4. Discussion

### 4.1. Principal Findings

To our knowledge, this is the first prospective study to longitudinally evaluate RNFL thickness and ONSD in pregnant women with and without preeclampsia using a combined high-resolution OCT and ocular ultrasound approach, while also exploring associations with angiogenic biomarkers such as the sFlt-1/PlGF ratio. RNFL thickness was generally higher in the preeclampsia group. After accounting for the correlation between both eyes using GEE, exploratory between-group differences in RNFL thickness were observed at visit 2, whereas differences at visits 1 and 3 did not reach statistical significance. ONSD did not differ significantly between groups at any visit. No significant between-group differences in longitudinal change from early to late postpartum were observed for RNFL thickness or ONSD.

### 4.2. RNFL Changes in Preeclampsia

Pregnancy induces physiological changes such as fluid retention and hormonal shifts, which can affect ocular tissues and visual function, potentially leading to both adaptations and exacerbation of underlying pathologies [20,21].

The literature on RNFL changes in preeclampsia remains heterogeneous, as highlighted by a recent systematic review of retinal imaging in preeclamptic pregnancy [22]. Prior studies have reported increased RNFL thickness in healthy pregnant women compared to non-pregnant controls, particularly in the nasal superior and nasal inferior quadrants. Wu et al. [23] attributed this to gestational vasodilation, whereas Demir et al. [24] did not observe significant differences in overall RNFL thickness. Some studies found no significant differences between preeclamptic and normotensive pregnant women, while others observed increased RNFL thickness in preeclamptic patients with abnormal retinal findings [21,25]. Additional data suggest that pregnancy itself may be associated with increased RNFL thickness compared with non-pregnant controls [26].

Our findings align with these previous observations, showing generally greater RNFL thickness in the preeclampsia group. After accounting for the paired-eye structure, an exploratory between-group difference was observed at the early postpartum visit, whereas differences at the antepartum and late postpartum visits did not reach statistical significance. The numerically greater RNFL thickness observed in women with early-onset preeclampsia, including one nominally significant site-specific difference that did not persist after FDR adjustment, should be considered exploratory and hypothesis-generating. In our cohort, no cases of clinical papilledema were documented, despite the observed differences in RNFL thickness. Whether these subtle RNFL differences reflect subclinical neuro-ophthalmologic involvement cannot be determined from the present data; however, OCT may provide a sensitive means of characterizing structural retinal changes not apparent on conventional fundoscopy. Unlike earlier studies that typically employed quadrant-based measurements (superior, inferior, nasal, and temporal), we used a zonal approach based on concentric rings (3.5 mm and 4.1 mm) around the optic disk to quantify RNFL thickness. This method offers anatomically standardized and reproducible assessments and may help highlight diffuse peripapillary changes that span across traditional quadrant boundaries. However, it may be less sensitive to localized focal defects compared to quadrant or sectoral analyses. While not necessarily superior, it represents a complementary approach to conventional quadrant analysis. Additionally, by summarizing RNFL thickness across entire rings, this approach may provide a quicker and more intuitive overview particularly for non-specialists or in interdisciplinary settings. This distinction is important when comparing our findings to earlier quadrant-based studies, as ring-based RNFL values are not directly interchangeable with quadrant values due to differing anatomical and topographic references.

### 4.3. ONSD in Preeclampsia

The literature on ONSD in preeclampsia is similarly inconclusive. A pilot study by Dubost et al. reported normal ONSD values in healthy pregnancies but elevated values (>5.8 mm) in 19% of preeclamptic cases [7,27]. However, validated reference values for pregnancy are lacking, and the optimal ONSD cutoff for elevated intracranial pressure remains uncertain. A South African study recently reported higher ONSD values in late-onset compared to early-onset preeclampsia, with 28% versus 3% of women exceeding the 5.8 mm threshold [28,29]. Preeclampsia is increasingly recognized as a heterogeneous syndrome with distinct cardiovascular phenotypes [30], and the observed ONSD differences may similarly reflect phenotype-specific variation, although their pathophysiological significance remains uncertain. Yet, as ONSD was measured only once antepartum in those studies, the temporal progression and clinical significance remain unclear.

In our study, transbulbar ultrasound did not reveal significant ONSD differences between groups at any time point. No participant had a bilateral mean ONSD > 5.8 mm at any time point. The absence of markedly elevated ONSD values may partly reflect the inclusion of an unselected preeclampsia population rather than a cohort enriched for neurological or visual symptoms.

### 4.4. Angiogenic Biomarkers and RNFL

In addition to structural imaging, alterations in angiogenic biomarkers are central to the pathophysiology of preeclampsia. Elevated sFlt-1 levels and reduced PlGF concentrations are associated with endothelial dysfunction and have been linked to changes in choroidal thickness [26,31]. While the effect of these angiogenic factors on RNFL thickness has not been clearly established, their role in vascular permeability suggests a potential relationship.

In a post hoc exploratory analysis, a nominally significant positive correlation was observed between RNFL thickness in the right eye at 4.1 mm and the sFlt-1/PlGF ratio in the preeclampsia group (ρ = 0.621, *p* = 0.024). However, this association did not remain significant after adjustment for multiple comparisons (FDR-adjusted q = 0.384) and was not observed at other measurement sites or in the control group. Although this finding may suggest a possible relationship between antiangiogenic imbalance and RNFL changes in preeclampsia, its isolated, site- and eye-specific nature and the lack of significance after adjustment for multiple testing warrant cautious interpretation. It should therefore be regarded as hypothesis-generating and may represent a chance finding. Confirmation in larger, adequately powered cohorts is required.

Most previous studies have evaluated OCT or ONSD separately and have predominantly been cross-sectional. Recent prospective OCT angiography (OCTA) data have begun to characterize peripartum changes in the retinal microvasculature [32]. The present study extends this literature by combining longitudinal OCT, ocular ultrasound, and angiogenic biomarkers across the peripartum period.

### 4.5. Limitations

This study has several limitations. The relatively small sample size and single-center design may limit the precision and generalizability of the findings, particularly with regard to heterogeneity across ethnicity, parity, and maternal comorbidity. As measurements from both eyes of each participant are correlated, the paired-eye structure was addressed using Generalized Estimating Equations with participant-level clustering; a sensitivity analysis using one randomly selected eye per participant (500 independent draws) supported this general pattern, although the proportion of draws reaching nominal significance was reduced compared with the GEE model, particularly at visit 1 and visit 3, and ONSD showed no significant difference in any draw at any visit (Supplementary Table S1). Given the limited number of participant-level clusters, particularly at visit 2, the robust sandwich variance estimator used in the GEE models may be somewhat anti-conservative in small samples; the borderline significance observed at visit 2 should therefore be interpreted with caution rather than as definitive evidence of a true group difference. Data availability was lower at the early postpartum visit (visit 2; approximately half of participants) than at the antepartum visit, and ONSD data availability was additionally reduced relative to RNFL at the late postpartum visit (visit 3: ONSD *n* = 9 vs. RNFL *n* = 12 in the preeclampsia group), which may have affected the representativeness of findings at these time points. In addition, multiple exploratory comparisons were made; FDR-adjusted q-values were therefore provided for the subgroup, correlation and BMI analyses, and these findings should be regarded as hypothesis-generating. The absence of a non-pregnant control group limits differentiation between physiological pregnancy-related and preeclampsia-related changes. As an exploratory pilot study, no formal a priori sample-size calculation was performed, and the study was not designed to establish diagnostic cut-off values. Nonetheless, all imaging was conducted by trained investigators using standardized protocols, and the prospective longitudinal design represents a strength of the study.

## 5. Conclusions

In conclusion, the findings support the presence of peripartum changes in RNFL thickness and ONSD, with RNFL alterations potentially being more pronounced in women with preeclampsia. However, ONSD did not clearly differentiate women with preeclampsia from normotensive pregnant controls, and associations with angiogenic biomarkers did not remain significant after adjustment for multiple testing. These findings should therefore be considered exploratory and hypothesis-generating. Their clinical relevance remains to be clarified in larger, adequately powered prospective multicenter studies, ideally with correlation to neurological and visual outcomes.

## Figures and Tables

**Table 1:** Maternal demographic and baseline characteristics.

	Preeclampsia (*n* = 15)	Control (*n* = 23)	*p*-Value
Age (y) Mean (SD)	30.1 (5.6)	32.3 (4.4)	0.294
BMI (kg/m^2^) Median [IQR]	30.5 [27.6–41.2]	28.2 [26.2–32.6]	0.124
ASA physical status			0.039
2	9 (60%)	21 (91%)	
>2	6 (40%)	2 (9%)	
Parity	0 [0–0.5]	0 [0–1]	0.282
Gravidity	1 [1–2]	2 [1–3]	0.217
Obstetric history			
GDM History of previous preeclampsia	3 (20%)	9 (39%)	0.294
	3 (20%)	4 (17%)	1.000
LDA prophylaxis	4 (27%)	8 (35%)	0.728

**Table 2:** Clinical, obstetric, and neonatal characteristics.

	Preeclampsia (*n* = 15)	Control (*n* = 23)	*p*-Value
Gestational age at delivery (weeks) Mean (SD)	34 + 2 (4.5)	38 + 6 (1.8)	<0.001
sFlt-1/PlGF ratio at screening (Preeclampsia *n* = 14; Control *n* = 21)	109.2 [64.6–280.4]	15.9 [5.1–29.0]	<0.001
Headache or visual disturbances	6 (40%)	0 (0%)	0.002
Mode of delivery			0.013
Vaginal	1 (7%)	13 (57%)	
Operative vaginal	3 (20%)	2 (9%)	
Primary CD	6 (40%)	3 (13%)	
Secondary CD	4 (27%)	4 (17%)	
Emergency CD	1 (7%)	1 (4%)	
1 min Apgar score Median [IQR]	8 [7.5–9]	9 [9–9]	0.017
5 min Apgar score Median [IQR]	9 [8–10]	10 [9–10]	0.110

## Data Availability

The original data generated for this study are available from the corresponding author upon reasonable request. The data are not publicly available because they contain clinical patient-level data from a small prospective cohort and are subject to institutional data-protection regulations, ethical restrictions, and participant privacy. If required, we would be willing to discuss the provision of a de-identified minimal dataset for internal editorial or peer-review evaluation, provided this is permitted under the applicable institutional data-protection and ethical regulations.

## Supplementary Materials

The following supporting information can be downloaded at: https://www.mdpi.com/article/10.3390/jcm15176618/s1, Table S1: Generalized Estimating Equations (GEE) analysis of RNFL thickness and ONSD group differences (Preeclampsia vs. Control), accounting for the paired-eye structure (participant-level clustering, exchangeable correlation, eye as covariate, robust standard errors); Table S2: Changes in RNFL thickness and ONSD measurements from visit 2 to visit 3; Table S3: Exploratory subgroup analysis of RNFL thickness, ONSD and angiogenic biomarker measurements in women with early- versus late-onset preeclampsia at visit 1; Table S4: Correlation between the sFlt-1/PlGF ratio and RNFL thickness and ONSD measurements at visit 1.
