# Supplementary material for: Longitudinal Peripartum Assessment of Retinal Nerve Fiber Layer Thickness and Optic Nerve Sheath Diameter in Women with Preeclampsia and Normotensive Pregnant Controls
Source: J Clin Med. 2026 Aug 27;15(17):6618. doi: 10.3390/jcm15176618 (PMC13565923; doi:10.3390/jcm15176618)
Supplement: Supplementary file 1 [file jcm-15-06618-s001.zip › jcm-4447328-supplementary.pdf]

Table S1: Generalized Estimating Equations (GEE) analysis of retinal nerve fiber layer (RNFL) thickness and optic nerve sheath diameter (ONSD) group differences (Preeclampsia vs. Control), accounting for the paired-eye structure (participant-level clustering, exchangeable correlation, eye as covariate, robust standard errors). Results of the one-eye sensitivity check are reported in the last column.

Measurement	Visit	Estimate (P vs. Control)	Robust SE	p value	% of 500 one-eye draws p<0.05
RNFL thickness (µm)					
3.5 mm	Visit 1	4.79	2.55	0.060	4.4%
	Visit 2	9.18	4.24	0.030	18.6%
	Visit 3	5.38	2.76	0.051	17.8%
	Δ (Visit 2 to 3)	-0.52	0.67	0.434	1%
4.1 mm	Visit 1	4.18	2.18	0.056	3%
	Visit 2	7.59	3.46	0.028	46.8%
	Visit 3	3.65	2.39	0.127	0%
	Δ (Visit 2 to 3)	-1.02	0.79	0.194	1.4%
ONSD (mm)					
Longitudinal	Visit 1	0.24	0.22	0.295	0%
	Visit 2	-0.03	0.35	0.939	0%
	Visit 3	0.08	0.22	0.721	0%
	Δ (Visit 2 to 3)	-0.12	0.39	0.751	0%
Transverse	Visit 1	0.12	0.26	0.648	0%
	Visit 2	0.15	0.34	0.656	0%
	Visit 3	-0.03	0.18	0.865	0%
	Δ (Visit 2 to 3)	0.004	0.27	0.987	0%

Estimates represent the group difference (Preeclampsia minus Control) from GEE models (Gaussian family, exchangeable working correlation, participant-level clustering, adjusted for eye). Δ (Visit 2 to 3) rows are based on participants with

complete paired data at both visits: RNFL n = 17 (10 control, 7 preeclampsia); ONSD n = 14 (9 control, 5 preeclampsia). RNFL = retinal nerve fiber layer, ONSD = optic nerve sheath diameter, SE = standard error.

Table S2: Changes in retinal nerve fiber layer (RNFL) thickness and optic nerve sheath diameter (ONSD) measurements from visit 2 to visit 3

Measurement	Total	Preeclampsia	Control	p-value
RNFL thickness (μm)				
3.5 mm left	0 (-1-1)	0 (-1.5-0.5)	0 (-0.75-1)	0.51
4.1 mm left	-1 (-1-0)	-1 (-1-0.5)	0 (-1-0.75)	0.18
3.5 mm right	-1.5 (-3.25-0)	-2 (-3.5-0.5)	-1 (-2-0)	0.48
4.1 mm right	-1 (-2-0)	-1 (-2.5-0.5)	-1 (-2-0.25)	0.8
ONSD (mm)				
Left L	-0.35 (-1.33-0.2)	-0.29 (-1.4-0.22)	-0.4 (-1.22-0.15)	0.79
Left T	-0.38 (-0.61-0.35)	-0.36(-0.39-0.32)	-0.44 (-0.61-0.44)	1
Right L	-0.24 (-1.01-0.36)	-0.24 (-0.66-0.17)	-0.22 (-1.21-0.61)	0.85
Right T	-0.33 (-0.74-0.32)	-0.33 (-0.59-0.01)	-0.22 (-1.01-0.32)	0.95

Data are presented as median change (Q1–Q3). RNFL = retinal nerve fiber layer, ONSD = optic nerve sheath diameter, L = longitudinal, T = transverse

Table S3: Exploratory subgroup analysis of retinal nerve fiber layer (RNFL) thickness, optic nerve sheath diameter (ONSD) and angiogenic biomarker measurements in women with early- versus late-onset preeclampsia at visit 1. q-values were calculated using the Benjamini-Hochberg procedure across all comparisons within this table.

Measurement	Total (n=15)	Early-onset (n=6)	Late-onset (n=9)	p-value	q-value (FDR)
RNFL thickness (μm)					
3.5 mm left	106 (99-109.25)	108.5 (105.25-114.5)	101.5 (96.5-107)	0.12	0.27
4.1 mm left	90.5 (84-94.75)	92 (88.25-98.75)	87.5 (80.75-92.5)	0.18	0.324
3.5 mm right	107 (99.78–114.25)	115.5 (106–117.5)	102.85 (98.75–108)	**0.045**	0.24
4.1 mm right	92 (83.5–95)	95.5 (89–97.5)	88.5 (82.75–92)	0.08	0.24
ONSD (mm)					
Left L	4.69 (4.27–5.27)	4.46 (4.30–4.65)	5.05 (4.39–5.43)	0.43	0.645
Left T	4.63 (4.56–5.51)	4.62 (4.57–5.12)	5.07 (4.07–5.72)	1	1.00
Right L	4.69 (4.41–5.20)	4.54 (4.42–4.99)	4.99 (4.35–5.31)	0.62	0.797
Right T	4.91 (4.39–5.12)	4.87 (4.48–5.07)	4.91 (4.07–5.27)	0.94	1.00
sFlt-1/PIGF ratio					
	109.2 (64.57-280.45)	350.8 (195.7-397.6)	95.6 (60.1-112)	0.06	0.24

Subgroup n-values refer to overall subgroup membership; imaging analyses were based on available measurements and therefore included fewer participants for individual modalities. Data are presented as median (Q1–Q3). sFlt-1/PIGF data available for 14 of 15 women with preeclampsia (early-onset n = 5; late-onset n = 9). RNFL = retinal nerve fiber layer, ONSD = optic nerve sheath diameter, L = longitudinal, T = transverse, FDR = false discovery rate.

Table S4: Correlation between the soluble fms-like tyrosine kinase-1/placental growth factor (sFlt-1/PlGF) ratio and retinal nerve fiber layer (RNFL) thickness and optic nerve sheath diameter (ONSD) measurements at visit 1. q-values were calculated using the Benjamini-Hochberg procedure across all 16 correlations reported in this table.

Variable	Group	Spearman's ρ	p-value	q-value (FDR)
RNFL thickness (μm)				
3.5 mm left	Control	-0.151	0.551	0.588
	Preeclampsia	0.396	0.181	0.516
4.1 mm left	Control	-0.256	0.290	0.516
	Preeclampsia	0.369	0.215	0.516
3.5 mm right	Control	-0.229	0.332	0.531
	Preeclampsia	0.481	0.096	0.516
4.1 mm right	Control	-0.332	0.153	0.516
	Preeclampsia	0.621	0.024	0.384
ONSD (mm)				
Left L	Control	-0.098	0.699	0.699
	Preeclampsia	0.196	0.543	0.588
Left T	Control	-0.267	0.282	0.516
	Preeclampsia	0.273	0.390	0.567
Right L	Control	-0.162	0.495	0.588
	Preeclampsia	0.217	0.499	0.588
Right T	Control	-0.256	0.275	0.516
	Preeclampsia	0.378	0.227	0.516

Correlations are reported as Spearman's ρ. q-values were calculated using the Benjamini–Hochberg procedure across all 16 correlations reported in this table. RNFL = retinal nerve fiber layer; ONSD = optic nerve sheath diameter; L = longitudinal; T = transverse; FDR = false discovery rate.
